# Overexpression of hepatocyte EphA2 enhances liver-stage infection by *Plasmodium vivax*

**DOI:** 10.1038/s41598-022-25281-4

**Published:** 2022-12-13

**Authors:** Sittinont Chainarin, Ubonwan Jaihan, Parsakorn Tapaopong, Pinyapat Kongngen, Nawapol Kunkeaw, Liwang Cui, Jetsumon Sattabongkot, Wang Nguitragool, Wanlapa Roobsoong

**Affiliations:** 1 Mahidol Vivax Research Unit, Faculty of Tropical Medicine, Mahidol University, Bangkok, 10400 Thailand; 2 Department of Molecular Tropical Medicine and Genetics, Faculty of Tropical Medicine, Mahidol University, Bangkok, 10400 Thailand; 3 Department of Internal Medicine, Morsani College of Medicine, University of South Florida, Tampa, FL 33612 USA

**Keywords:** Gene expression, Malaria

## Abstract

The liver is the first destination of malaria parasites in humans. After reaching the liver by the blood stream, *Plasmodium* sporozoites cross the liver sinusoid epithelium, enter and exit several hepatocytes, and eventually invade a final hepatocyte host cell. At present, the mechanism of hepatocyte invasion is only partially understood, presenting a key research gap with opportunities for the development of new therapeutics. Recently, human EphA2, a membrane-bound receptor tyrosine kinase, was implicated in hepatocyte infection by the human malaria parasite *Plasmodium falciparum* and the rodent parasite *Plasmodium yoelii*, but its role is not known for *Plasmodium vivax*, a major human parasite whose liver infection poses a specific challenge for malaria treatment and elimination. In this study, the role of EphA2 in *P. vivax* infection was investigated. It was found that surface expression of several recombinant fragments of EphA2 enhanced the parasite infection rate, thus establishing its role in *P. vivax* infection. Furthermore, a new permanent cell line (EphA2Extra-HC04) expressing the whole extracellular domain of EphA2 was generated. This cell line supports a higher rate of *P. vivax* infection and is a valuable tool for *P. vivax* liver-stage research.

## Introduction

Malaria parasites are spread by female *Anopheles* mosquitoes, which carry the sporozoites in the salivary glands. Human malaria infection begins when *Plasmodium* sporozoites are deposited in the dermis during mosquito bites. These sporozoites then migrate to the blood circulation and are transported to the liver. Once in the liver, the parasites leave the liver sinusoid, by breaching the sinusoidal cell layer^1,2^ or penetrating the Kupffer cells^3^ to gain access to the hepatocytes. After the successful invasion of a hepatocyte, each sporozoite develops to the liver-stage (LS), also called the exoerythrocytic forms (EEFs), a process which is completed in approximately one week, resulting in thousands of merozoites ready to cause blood infection and clinical disease.

Although liver infection by malaria parasites is a key target of malaria prophylactic drug and vaccine development^4^, the understanding of how *Plasmodium* sporozoites recognize and infect hepatocytes is still limited. To establish infection in the liver, sporozoites penetrate and traverse multiple hepatocytes before finally invading the hepatocyte host cell^5,6^. The invasion process is characterized by the formation of a parasitophorous vacuole that encloses the intracellular parasite. Hepatocyte invasion relies on a few known parasite proteins, which interact with host receptors on the hepatocyte surface^4^. The P36 and P52 complex is required for productive invasion and maintenance of the liver-stage parasites^4,7–9^. During in vitro invasion of *P. yoelii*, a rodent malaria parasite, P36 is discharged upon sporozoite activation and interacts with P52 as a complex to engage host receptors^9^. While the role of P52 appears conserved across different *Plasmodium* species, the species-specific host receptor for *P. yoelii* and *P. berghei* is predominantly determined by P36^7^.

Three human hepatocyte receptors have been identified for sporozoite invasion—tetraspanin (CD81), Scavenger Receptor class B type I (SR-BI), and Ephrin type-A receptor 2 (EphA2). These proteins have been implicated in *P. falciparum*^7,10–13^, *P. yoelii*^7,10,11,13–15^, and *P. berghei*^7,12,15,16^, although receptor utilization appears to vary among different studies^7,10–16^, presumably reflecting redundant invasion pathways used by the parasites. For *P. vivax*, SR-BI has been the only host protein with an established function in hepatocyte infection^7^. Antibodies against this protein significantly inhibit *P. vivax* infection of human primary hepatocytes. However, the inhibition is incomplete, suggesting involvement of one or more additional receptors. EphA2 is a possible candidate because previous studies have demonstrated its role for *P. yoelli* and *P. falciparum*^13^. This protein may thus be a receptor for *P. vivax*. Here, the role of EphA2 in *P. vivax* hepatocyte infection was explored. Mammalian cell transfection was used to induce the expression of six different partial and full-length constructs of EphA2 on the surface of HC-04^17^. The susceptibility of these transfected cells to *P. vivax* infection was evaluated.

## Results

### Recombinant EphA2 constructs and expression in HC-04

**Figure 1:**
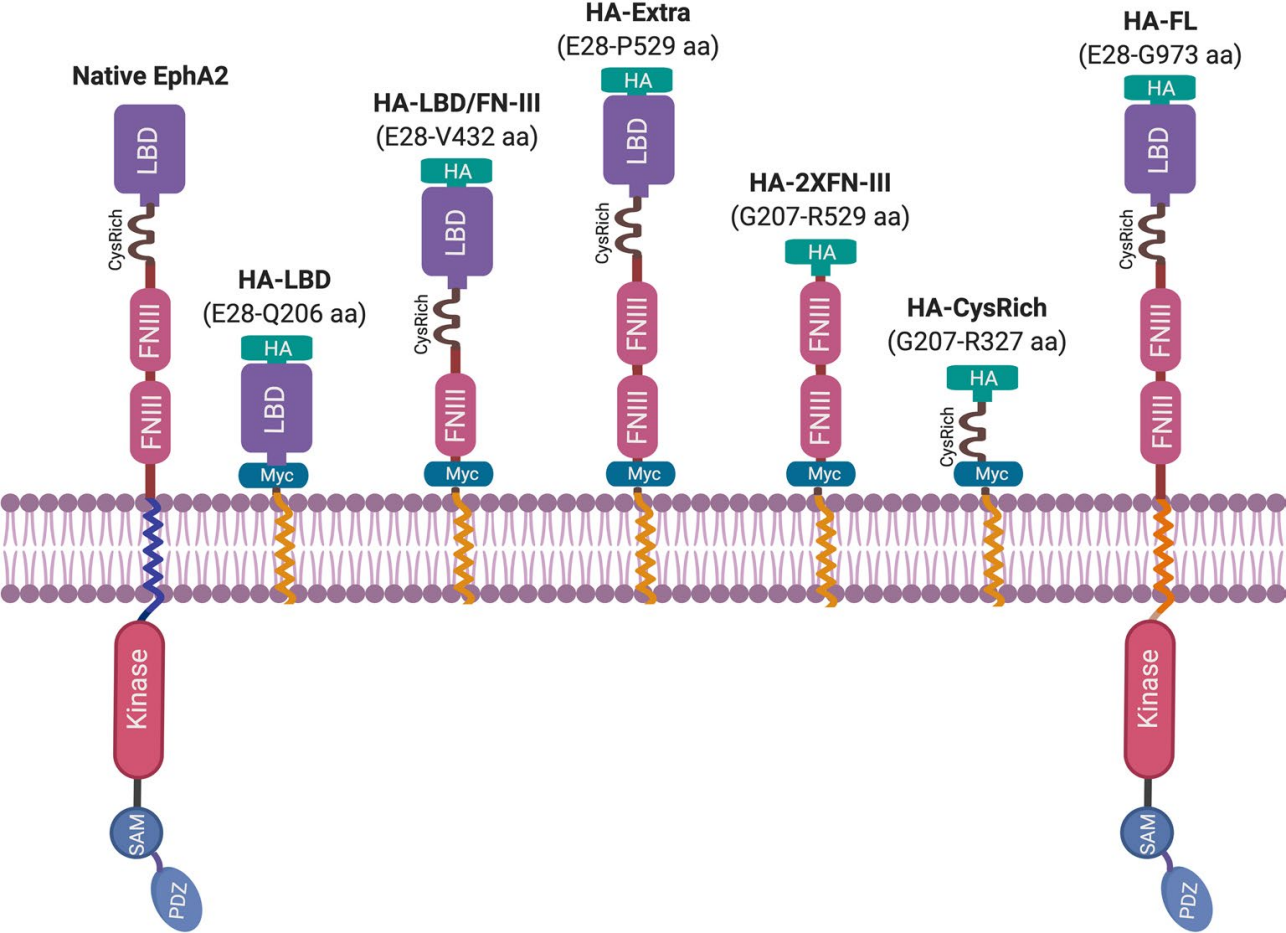
Schematic illustrations of recombinant EpA2 constructs. *HA:* HA-tag, *myc* myc-tag, *LBD:* ligand binding domain, *CysRich:* cysteine-rich domain, *FNIII:* fibronectin III repeat, *Kinase:* kinase domain, *SAM:* sterile α-motif domain, *PDZ:* PDZ-binding motif. The native TMD of EphA2 is depicted in blue; the PDGFR TMD is in orange. The figure was created with BioRender.com.

**Figure 2:**
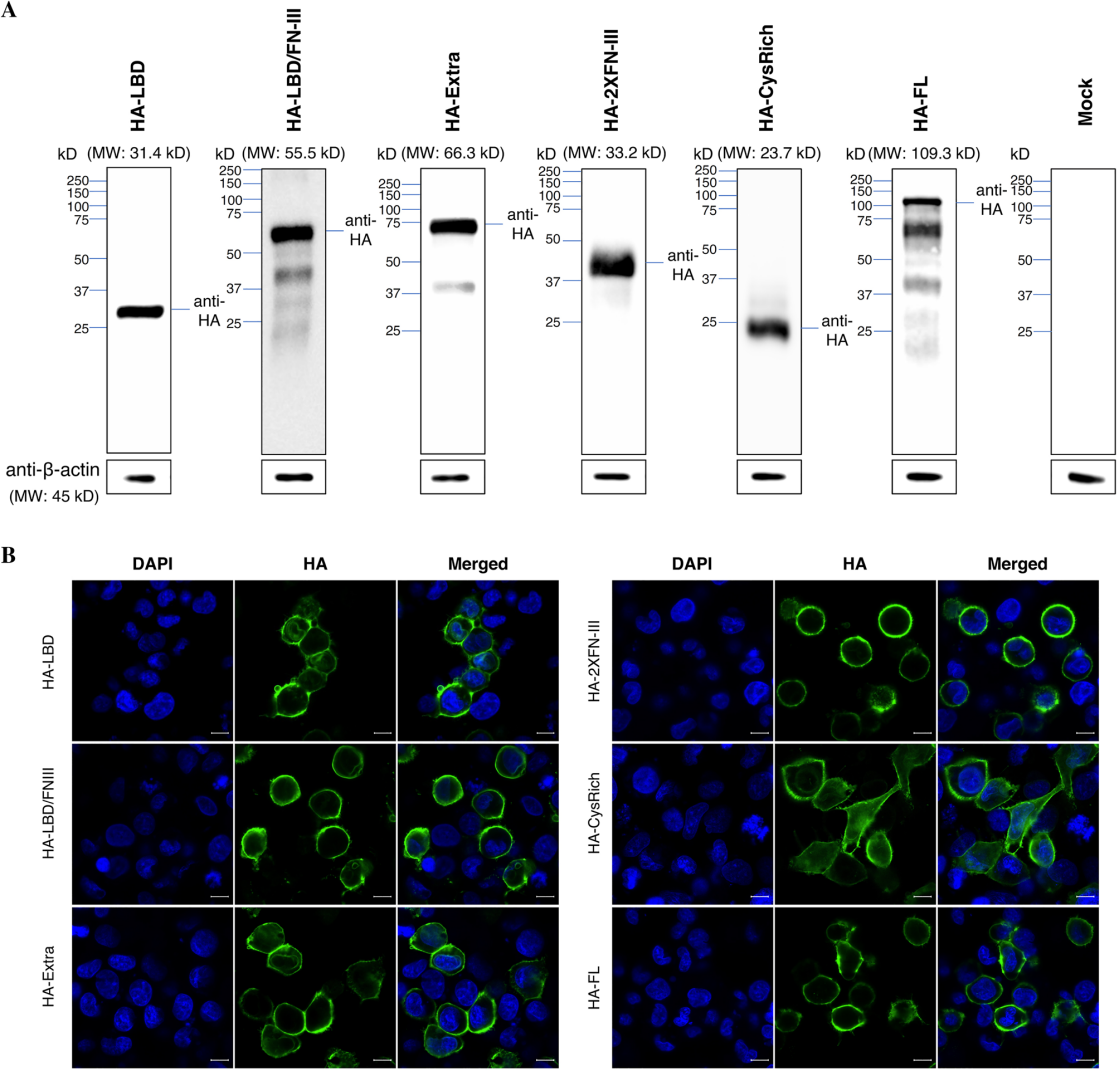

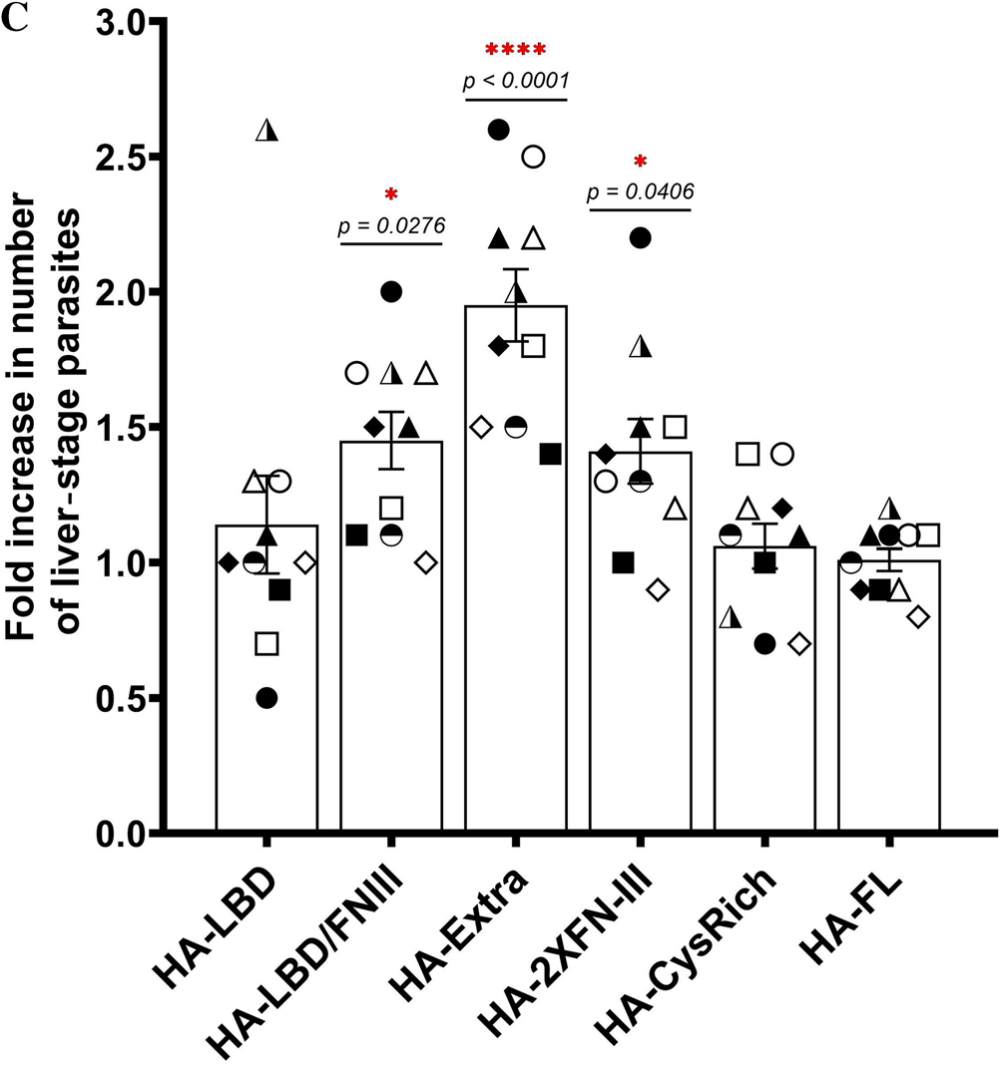
Transient expression of EphA2 constructs in HC-04 and the impact on *P. vivax* infection. (**A**) Western blot of the total cell lysates from HC-04 transfected with the indicated EphA2 construct. The EphA2 recombinant proteins were detected through the N-terminal HA-tag. These blots are not quantitative as different exposure were used to achieve best visual clarity. The β-actin control bands were cropped from different gels using the same respective cell lysis. (**B**) Representative IFA images of non-permeabilized HC-04 complemented with each EphA2 construct. EphA2 recombinant protein was detected by an HA-tag antibody (green). Nuclei (blue) were visualized with DAPI. Scale bar: 10 μm. (**C**) Fold increase of the liver-stage parasite infection in HC-04 expressing different EphA2 constructs. The numbers of liver-stage parasites were quantified by immunostaining with UIS4 antibody on day 4 post-infection. Error bars represent S.E.M. of 10 *P. vivax* clinical isolates. Different symbols represent different *P. vivax* clinical isolates. Asterisks mark significant differences from the mock transfection control; *p*-values were determined by two-way ANOVA (EphA2 construct and parasite isolate as variables) followed by Dunnett’s multiple comparisons test.

EphA2 (Fig. 1) is a 976 amino acid-long single-transmembrane human protein present in the plasma membrane of cells. Its extracellular N-terminal portion consists of a conserved ligand-binding domain (LBD) followed by a cysteine-rich (CysRich) domain, and two fibronectin III (FN-III) repeats that are linked to the transmembrane domain (TMD). The cytoplasmic C-terminal portion following the TMD contains a kinase domain, a sterile α-motif domain (SAM), and a PDZ-binding motif^18^. To investigate the role of EphA2 in *P. vivax* sporozoite infection of hepatocytes, we generated six recombinant EphA2 constructs (Fig. 1), each having a distinct domain composition. These constructs were designed for surface expression using the pDisplay™ mammalian expression vector^19^. All constructs carry an N-terminal HA-tag, which enables immuno-fluorescence staining. Except for the full-length construct (HA-FL), all constructs are anchored to the plasma membrane by the TMD of the platelet-derived growth factor receptor (PDGFR) which is the default TMD of the pDisplay™ vector. The full-length construct, HA-FL, on the other hand, was generated by cloning the full-length gene (without the signal peptide) into the pDisplay™ plasmid with a stop codon before the PDGFR TMD coding sequence, resulting in a construct that is nearly identical to the endogenous EphA2. After transfection of the HC-04 cell line with these plasmids, the expression of each recombinant protein was confirmed by Western blot (Fig. 2a). Immuno-fluorescence assays (IFAs) against the extracellular HA-tag performed without cell-permeabilization showed a peripheral pattern of protein expression for all constructs, confirming their presence on the plasma membrane (Fig. 2b). The transfection efficiency was 20–30% as measured by flow cytometry (Supplementary Fig. S1).

### Expression of recombinant EphA2 can enhance *P. vivax* infection of HC-04

To determine the effect of each EphA2 construct on *P. vivax* infection, plasmids were freshly transfected into the HC-04 cell line 24 h before initiating the hepatocyte infection assay. Therefore, for each sporozoite batch, new transfection was performed. Ten different clinical isolates of *P. vivax* were tested, and the highest number of liver-stage parasites was observed in HC-04 transfected with the HA-Extra construct (Fig. 2c, Supplementary Table S1). This construct induced on average a twofold increase in the infection rate relative to the mock transfection control with the empty pDisplay™ plasmid. Two other constructs, HA-LBD/FN-III and HA-2XFN-III, also led to a significant but less pronounced increase. Lastly, cells transfected with the CysRich and LBD constructs did not differ in their susceptibility to *P. vivax* relative to the mock control. These results suggest that the extracellular domain of EphA2 is functional for *P. vivax* infection without the endogenous TMD and the cytoplasmic portion, and that larger extracellular constructs appeared to have a more significant impact on sporozoite invasion. Interestingly, no enhancement was detected when the full-length construct (HA-FL) was used. This could be due to protein truncation or degradation, as indicated by the presence of multiple bands on Western blot (Fig. 2a).

### Generation of a new transgenic HC-04 cell line with improved *P. vivax* susceptibility

**Figure 3:**
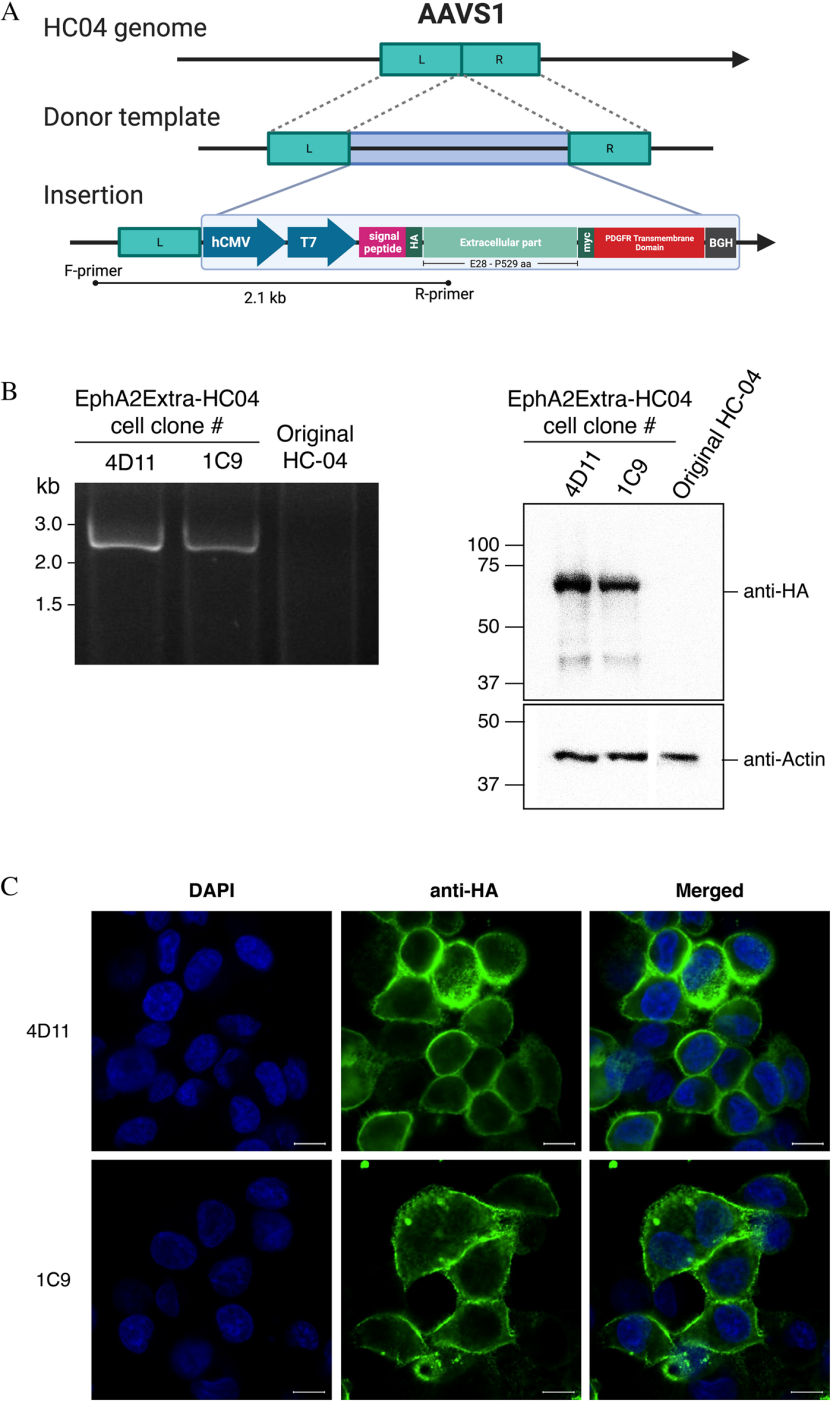
Generation of the transgenic EphA2Extra-HC04 cell line using a CRISPR-Cas9 knock-in approach. (**A**) Introduction of HA-Extra to the HC-04 genome by Cas9-mediated homology directed repair (HDR). Genome integration was induced by Cas9 with sgRNA targeting the AAVS1 sequence. Homologous recombination was achieved using a linear donor containing the HA-Extra cassette flanked by AAVS1 homology arms. The figure was created with BioRender.com. (**B**) *Left:* PCR demonstrating the presence of the HA-Extra sequence in two clones of the transgenic EphA2Extra-HC04 cell line. The forward primer binds to a sequence upstream of the left homology arm; the reverse primer binds to the ligand-binding domain (LBD) of the insert, resulting in a 2143-bp amplicon. Primer binding sites are depicted in (**A**). *Right:* Western blot analysis of the EphA2Extra-HC04 cell line. Total cell lysates were analyzed with the indicated antibodies. β-actin was used as the loading control. (**C**) Representative IFA images of EphA2Extra-HC04 clones expressing HA-Extra on the cell surface. HA-Extra was visualized with a HA-tag antibody. Nuclei were stained with DAPI. Scale bar: 10 μm.

**Figure 4:**
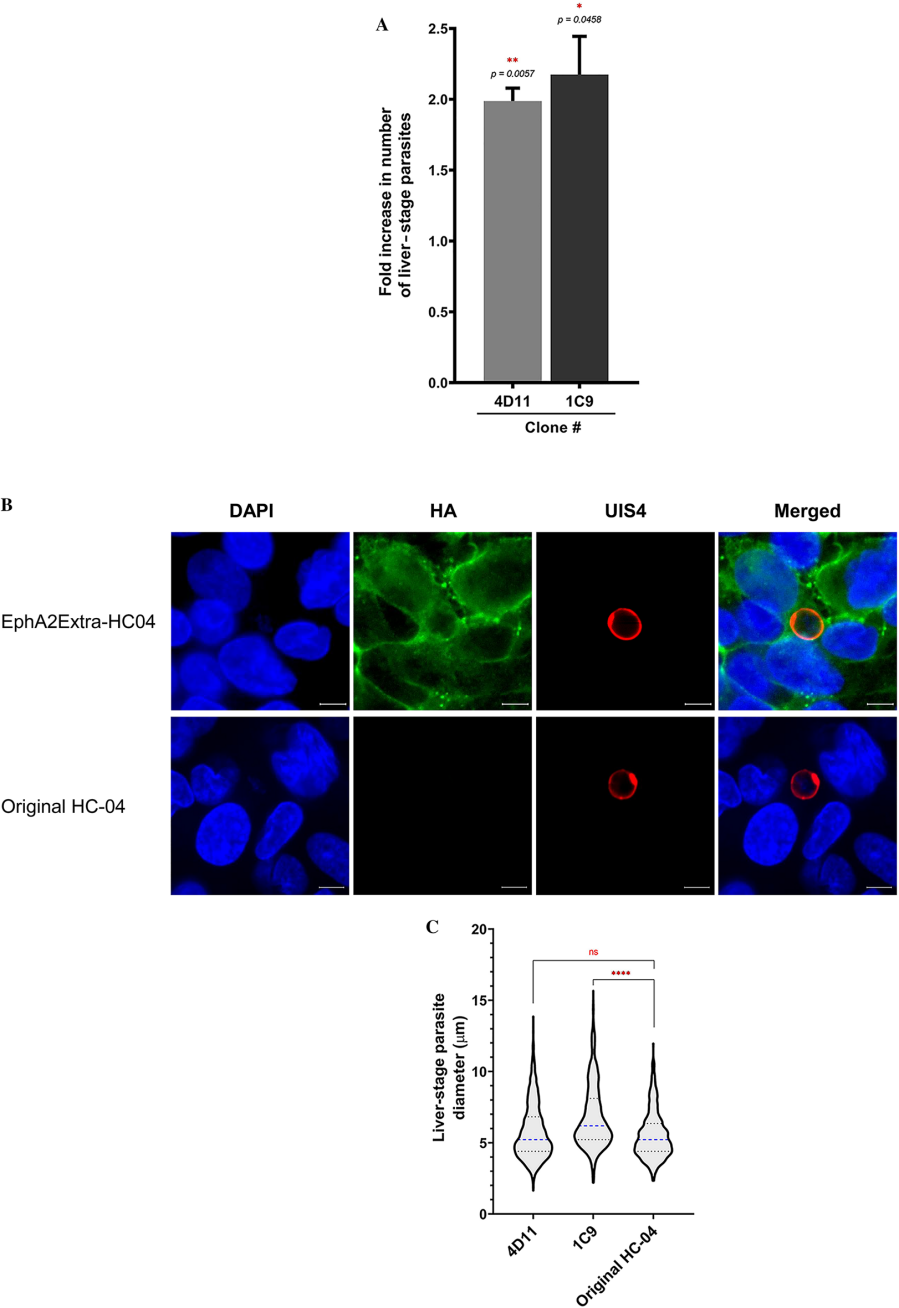
EphA2Extra-HC04 cells enhanced *P. vivax* liver-stage infection. (**A**) Fold increase of *P. vivax* infection of the two EphA2Extra-HC04 clones relative to the original HC-04. Error bars represent S.E.M. of 8 biological replicates. The *p*-value for two-tail paired t-test between EphA2Extra-HC04 cell clones and original HC-04. (**B**) *P. vivax*-infected EphA2Extra-HC04 (top) and original HC-04 (bottom) were labeled with a HA-tag antibody (green). Liver-stage parasites were labeled with a UIS4 antibody (red). Nuclei were stained with DAPI (blue). The scale bar represents 5 μm. (**C**) The size distribution of liver-stage parasites in each cell line. The diameters of intrahepatic parasites on day 4 post-infection were pooled from eight *P. vivax* clinical isolates. Dashed lines represent the medians; dotted lines delimit the interquartile ranges. ****Indicates a *p*-value < 0.0001 and ns indicates a non-significant test result (*p* = 0.0761) by the Mann–Whitney test between the indicated pair.

The in vitro study of *P. vivax* liver-stage development is highly challenging due to several factors, from sporozoite production, which requires a robust mosquito colony and fresh blood from *P. vivax* malaria patients, to the extremely low sporozoite infection rates in hepatic cell lines^17,20,21^. Given the enhancement of infection achieved by the transient transfection above, we sought to improve the HC-04 cell line for *P. vivax* research. Using a CRISPR/Cas9 system, we successfully inserted the HA-Extra construct into the genome of HC-04 at the AAVS1 integration site (Fig. 3a). Two clones of the transgenic cell line (EphA2Extra-HC04), 4D11 and 1C9, were obtained. Integration and protein expression were confirmed by PCR and Western blot, respectively (Fig. 3b). IFAs showed circumferential labeling of the HA-tag, confirming proper protein trafficking (Fig. 3c). Like in the transient transfection experiment (Fig. 2b), these EphA2Extra-HC04 transgenic clones offered approximately twofold higher infection rates for *P. vivax* sporozoites than the original HC-04 cell line (Fig. 4a, Supplementary Table S2). Figure 4b shows representative parasites on day 4 post infection in EphA2Extra-HCO4 and original HC-04. The size distribution of day 4-parasites in the EphA2Extra-HCO4 clones and the original HC-04 were shown (Fig. 4c, Supplementary Fig. S2). At this timepoint, the parasites were generally smaller than 15 µm and too early to be size-differentiated into growing schizonts and hypnozoites. IFAs also revealed nuclear division (Supplementary Fig. S3). Together these data indicate that EphA2Extra-HC04 supports normal parasite development.

## Discussion

The research on *Plasmodium* hepatocyte infection and development has been severely limited relative to the research on the blood stages. *Plasmodium* sporozoites recognize a few hepatocyte surface proteins during invasion. Among these proteins is EphA2, a receptor tyrosine kinase, first identified as a *P. yoelii* and *P. falciparum* receptor through antibody array screening^13^. In our study, we investigated and demonstrated a functional role of the extracellular domain of EphA2 in *P. vivax* liver-stage infection, using an in vitro culture system of the hepatic HC-04 cell line. Through transient transfection of plasmids encoding different EphA2 constructs, we could achieve 20–30% transfection efficiency. Despite the low efficiency, the full extracellular construct (HA-Extra), which contains LBD, CysRich, and two FN-III repeats, could enhance the *P. vivax* infection two-fold. Smaller constructs containing LBD and FNIII or two FNIII’s could also promote infection, but to a smaller extent. In contrast, LBD or CysRich alone was not sufficient. Therefore, larger constructs of EphA2 tend to perform better. The reason for this is unclear, but it is possible that the larger constructs may increase the accessibility to the binding site due to its increased protrusion from the cell surface. It is also possible that there are more binding sites for the parasite ligand on the larger proteins. EphA2 is known to have multiple binding sites on different extracellular subdomains for its natural ligand EphrinA1^22–24^. Of note, the full-length construct did not promote productive invasion. It is possible that the native EphA2 TMD or the cytoplasmic portion present in the full-length protein hinders the interaction of EphA2 and its parasite ligand, perhaps by affecting the quaternary structure of EphA2. The SAM domain in the C-terminal half of EphA2 has been shown to inhibit the protein’s dimerization^25^. The molecular interaction between hepatocyte EphA2 and parasite ligand(s) awaits further investigation.

Because the transient transfection of EphA2 extracellular domains could enhance *P. vivax* infection at 20–30% transfection efficiency, we thought that higher infection rates could be obtained through stable transfection. Therefore, we used the CRISPR/Cas9 technology to introduce the HA-Extra construct to the genome of HC-04. However, to our surprise, this new cell line (EphA2Extra-HC04) was still only two-fold more susceptible to *P. vivax* than the original HC-04. It is thus possible that because sporozoites are motile, they can search for a suitable host cell, in which case increasing the proportion of suitable cells by genome integration would not lead to a further enhancement. Additionally, some parasites may not depend strongly on EphA2 if there is an alternative invasion pathway utilizing a different receptor protein.

Although the twofold increase in *P. vivax* susceptibility of EphA2Extra-HC04 is modest, it is highly valuable for *P. vivax* exoerythrocytic stage research. The number of sporozoites has been the single most important limiting factor for research of *P. vivax* pre-erythrocytic stages. By reducing the number of sporozoites by half to achieve the same invasion rate, researchers can broaden compound screening for drug development and test more antibodies to identify new vaccine targets. It is possible that overexpressing EphA2 with other host receptors such as SR-BI^7^ could further promote of *P. vivax* liver infection.

Our findings further suggest that *P. vivax* liver-stage development could be supported in vitro by EphA2Extra-HC04 cells. The size distribution of parasites in the engineered cell line was similar (clone 4D11) or greater (clone 1C9) to that of the parasites in the original HC-04 cell. There was also evidence of parasite nuclear division. However, due to the small size of the parasite at the early timepoint of parasite examination (4 days after inoculation), we were not able to reliably differentiate the growing schizonts from the dormant hypnozoites. Therefore, it remains unclear whether the introduction of EphA2-Extra would perturb the growing-schizont: hypnozoite ratio.

In summary, this study provides the first evidence supporting the role of EphA2 in *P. vivax* infection of hepatocytes. It suggests that EphA2 may be a common hepatocyte receptor across rodent and human malaria. The study also established a new cell line that supports a superior *P. vivax* infection rate. This new cell line is valuable for vaccine development, antimalarial compound screening, and fundamental research into parasite biology.

## Methods

### Construction of recombinant EphA2 fragments

Six recombinants of EphA2 (NM_004431.5) fragments (Fig. 1) were designed as a fusion protein with an N-terminal HA-tag. Gene fragments were PCR amplified from cDNA of HC-04 cell line. The primer sequences are listed in Supplementary Table S3. The thermal cycle was programmed as follows (i) 30 s at 98 °C for initial denaturation, (ii) 35 cycles of 15 s at 95 °C for denaturation, 30 s at 60 °C for annealing, and 120 s at 72 °C for extension, and (iii) 5 min at 72 °C for final extension. The amplified fragments were cloned into the pDisplay™ vector (Cat#V66020, Invitrogen) between the BglII and SalI restriction sites by the standard cloning technique.

### Hepatocyte culturing and transfection

HC-04 cells (Cat#MRA-975, BEI resources) were maintained in MEM-F12 medium (1:1) (Cat#41500034 & Cat#21700075, Gibco), supplemented with 20 mM NaHCO_3_, 15 mM HEPES, 10% (v/v) fetal bovine serum (Cat#10270106, Gibco), and 1% (v/v) Penicillin/Streptomycin (Cat#15140122, Gibco). Cells were kept in a humidified incubator at 5% CO_2_ and 37 °C. For transfection with recombinant EphA2 plasmids, HC-04 cells were plated at 5 × 10^4^ cells/well of a 96-well plate (Cat#655090, Greiner Bio-One) for 24 h prior to transfection. The transfection reaction for each well was prepared by mixing 400 ng of recombinant plasmid with 0.6 µL Lipofectamine™ 3000 and 1.6 μL P3000 (Cat#L3000015, Invitrogen) with the final volume adjusted to 10 μL with Opti-MEM® medium (Cat#31985062, Gibco) before transferring to each culture well. The culture plate was incubated for 24 h under humidified 5% CO_2_ and 37 °C. The expression of recombinant EphA2 on the surface of HC-04 was examined by Immunofluorescence staining and flow cytometry.

### Sporozoite production and hepatocyte infection

Blood was collected from *P. vivax* infected patients under the approved protocol by the Ethics Committee of the Faculty of Tropical Medicine, Mahidol University (MUTM 2018-016-02) and The Ministry of Public Health Ethical Review Committee of Research in human volunteer (Ref. No. 12 54-510). Informed consent was obtained from each patient before sample collection. The research was conducted in accordance with the Declaration of Helsinki.

Female *Anopheles dirus* were fed on *P. vivax* blood through membrane feeding as previously described^26^. Blood was collected from *P. vivax* patients during October, 2017–February, 2020. Each week a new batch of mosquitoes were produced for the experiments. Only *An. dirus* that fed on *P. vivax* mono-infection blood was used in the study. *P. vivax* sporozoites were harvested from salivary glands of the infected *An. dirus* on day 14–21 post feeding. Briefly, the mosquitoes were subjected to surface cleansing by 4-step sequential dipping: (i) 70% (v/v) ethanol, (ii) 10% (v/v) penicillin/streptomycin in sterile water, (iii) 25 μg/mL fungizone in sterile water, and (iv) RPMI1640 incomplete medium containing 1% (v/v) Pen/Strep, pH 8.0 (dissecting medium). The mosquito salivary glands were harvested under a stereomicroscope and kept in a 1.5 mL microcentrifuge tube containing 50 µL of cold dissecting medium. The salivary glands were washed once with dissecting medium and centrifuged at 13,000×*g* for 30 s. The salivary glands were resuspended in 50 µL of dissecting medium and the sporozoites were released by using a sterile pestle. The sporozoites were counted using a hemocytometer and diluted to 5 × 10^5^ sporozoites/mL. The culture supernatant of the transfected HC-04 was replaced with 100 µL of sporozoite suspension which yielded the final of 5 × 10^4^ sporozoites/well. The culture was maintained at 37 °C under 5% CO_2_ with daily change of culture medium. The parasite culture was harvested on day 4 post infection by fixing with 4% (w/v) paraformaldehyde for 30 min at the room temperature. The immunofluorescence staining was performed for quantification of liver-stage parasites.

### Generating a new permanent HC-04 cell line expressing HA-Extra

The HC-04 cell line was used to generate the transgenic HC-04 (EphA2Extra-HC04) cell line. The Cas9 plasmid, pSpCas9(BB)-2A-Puro (PX459) V2.0, was obtained from Addgene (Plasmid #62988). The sgRNA targeting the human AAVS1 gene, 5’-GATCGTGCGTCAGTTTTACCTGTGG-3’, was cloned into the plasmid. To prepare the linear donor DNA template, the entire HA-Extra expression cassette (under CMV promoter) flanked by 500 bp homology arms complementary to the AAVS1 target site was first synthesized and cloned into a plasmid. The linear template for transfection was then generated by PCR. The purified Cas9 plasmid and the linear donor template were transfected using Lipofectamine® 3000 reagents by mixing 500 ng plasmid DNA, 1500 ng linearized donor template DNA, 5 μL Lipofectamine® 3000 reagents, and 5 μL P3000 reagent in 50 μL Opti-MEM® medium. The transfection mixture was incubated at room temperature for 15 min. The transfection mixture was then transferred to 150,000 HC-04 cells in one well of a 24-well plate. Transfected cells were selected after 24 h post-transfection using 2 μg/μL puromycin (Cat#ant-pr-1, InvivoGen). Puromycin was maintained for 48 h. Fluorescence-activated cell sorting (FACS) to select HA-tag positive fluorescent cells was performed 20 days after transfection. Briefly, cells were detached using StemPro™ Accutase™ cell dissociation reagent (Cat#A1110501, Life sciences). Cell suspension was incubated in sterile-3% (w/v) bovine serum albumin (BSA) (Cat#A9418, Sigma Aldrich) in HC-04 incomplete medium for 30 min. Live cell immunofluorescence staining was performed by incubating HA-tag (6E2) mouse mAb (Cat#2367, Cell Signaling Technology) in HC-04 incomplete medium at 37 °C for 30 min. Suspension cells were washed three times using the HC-04 incomplete medium. Sorted cells were maintained under HC-04 growing conditions. A second round of FACS was performed 20 days after the first FACS to further enrich integrated cells. Lastly, limiting dilution was performed 48 h after the second FACS. Junction PCR was performed to determine the integration. Briefly, genomic DNA was extracted with 50 mM Tris (pH 8.0), 1 mM EDTA (pH 8.0), 0.5% (v/v) Tween 20, and > 0.0018 U/μL proteinase K at 37 °C for 18 h, followed by heat inactivation of proteinase K at 95 °C for 10 min^27^. PCR primers were designed to bind upstream of the left homology arm in the genomic locus and inside the LBD of the EphA2 (Supplementary Table S4). The thermal cycle was programmed as follows (i) 30 s at 98 °C for initial denaturation, (ii) 35 cycles of 15 s at 95 °C for denaturation, 30 s at 60 °C for annealing, and 120 s at 72 °C for extension, and (iii) 5 min at 72 °C for final extension.

### Immunofluorescence assays

For detection of EphA2, transfected or transgenic cells were fixed with 100 µL of 4% (w/v) paraformaldehyde (Cat#158127, Sigma Aldrich) in phosphate buffer saline (PBS) pH 7.4 for 30 min at the room temperature. After washing 3 times with 200 µL of PBS, cells were blocked in 200 µL of 3% (w/v) BSA/PBS (Cat#A9418, Sigma Aldrich) for 1 h. Cells were further incubated overnight at 4 °C with 100 µL of staining mixture containing Alexa Fluor-488 conjugated anti-HA-tag (6E2) mouse mAb (Cat#2350, Cell Signaling Technology) at 1:2000 dilution and 1 ng/mL DAPI and washed 3 times with 200 µL of PBS.

For liver-stage parasites, infected HC-04 cells were fixed with 100 µL of 4% (w/v) paraformaldehyde/PBS for 30 min at the room temperature and washed 3 times with 200 µL of PBS. Infected HC04 cells were permeabilized with 100 µL of 1% (v/v) triton X-100/PBS for 30 min and washed three times with 200 µL of PBS for 5 min each. Infected HC04 cells were incubated with 200 µL of 3% (w/v) BSA/PBS. Liver-stage parasites were stained with the staining mixture containing a mouse anti-UIS4 (1:4,200) monoclonal antibody (gift from Noah Sather) directly conjugated with Alexa Fluor® 488 (Cat#A20181, Invitrogen) and DAPI (1/1,000) (Cat#2350, Cell Signaling Technology) at the room temperature overnight (16 h), then washed 3 times with PBS.

For imaging, localization of recombinant EphA2 fragments and transgenic EphA2 in HC04 cells as well as liver-stage parasites were acquired using a Zeiss LSM-700 laser scanning confocal microscope. The numbers of liver-stage parasites were quantified using an Olympus IX73 inverted microscope as determined by UIS4 and DAPI.

### Western blotting

HC-04 cells solubilized in NuPAGE™ LDS sample buffer (Cat#NP0008, Invitrogen) were separated on 10% Tris–glycine SDS–polyacrylamide gels. Proteins were transferred onto the Immobilon-P polyvinylidene fluoride membrane (Cat#IPVH00010, Millipore). The membrane was blocked with 5% (w/v) skim milk in Tris Buffer Saline with Tween-20 (TBST). Proteins were probed with the primary HA-tag (6E2) mouse mAb (1: 2,500) (Cat#2367, Cell Signaling Technology) and anti-beta actin antibodies (1:10,000 for the loading control; Cat#ab8226, Abcam) in 5% (w/v) skim milk/TBST, washed three times, probed with horseradish peroxidase-conjugated secondary antibodies (1:2,000; Cat#12-349, Millipore), and developed using Immobilon Western chemiluminescent HRP substrate (Cat#WBKLS0500, Millipore).

## Supplementary Information

Supplementary Figures.

Supplementary Tables.

## Data Availability

The sequence of human EphA2 is publicly available on the NCBI database (Accession Number NM_004431.5, https://www.ncbi.nlm.nih.gov/nuccore/NM_004431.5). The liver-stage infection datasets generated and/or analyzed during the current study are available upon request and will be provided by the corresponding author W.R.
