# Supplementary material for: Overexpression of hepatocyte EphA2 enhances liver-stage infection by Plasmodium vivax
Source: Sci Rep. 2022 Dec 13;12:21542. doi: 10.1038/s41598-022-25281-4 (PMC9746569; doi:10.1038/s41598-022-25281-4)

Overexpression of hepatocyte EphA2 enhances liver-stage infection by *Plasmodium vivax*

Sittinont Chainarin[1,2], Ubonwan Jaihan[1], Parsakorn Tapaopong[1,2], Pinyapat Kongngen[1], Nawapol Kunkeaw[1], Liwang Cui[3], Jetsumon Sattabongkot[1], Wang Nguitragool[1,2*] & Wanlapa Roobsoong[1*]

[1]Mahidol Vivax Research Unit, Faculty of Tropical Medicine, Mahidol University, Bangkok, Thailand, 10400.

[2]Department of Molecular Tropical Medicine and Genetics, Faculty of Tropical Medicine, Mahidol University, Bangkok, Thailand, 10400.

[3]Department of Internal Medicine, Morsani College of Medicine, University of South Florida, Tampa, FL, 33612, USA.

* Corresponding authors: wanlapa.ros@mahidol.edu, wang.ngu@mahidol.edu

Supplementary figure S1. Representative transfection efficiency of recombinant EphA2 fragments. Error bars represent SEM. The surface expression of different domain of EphA2 during sporozoite invasion was quantified at 4 h after adding the sporozoites. The transfected HC-04 cells were detached using StemPro™ Accutase™ cell dissociation reagent and pelleted at 600 ×g for 5 min before washing with 1x Dulbecco's phosphate buffer saline (DPBS). The cell suspension was fixed with 4% (w/v) paraformaldehyde/PBS and 0.1 % (v/v) glutaraldehyde in the total volume of 0.5 ml for 30 min at the room temperature, and washed with DPBS twice. Suspended cells were incubated with 0.5 mL of 3% (w/v) bovine serum albumin (BSA) for 60 min at the room temperature. Quantitative detection of surface recombinant EphA2 was made using 1 μL of Alexa Fluor 488- conjugated HA-tag (6E2) mouse mAB (Cat#2350, Cell Signaling Technology) for 1.5× 10^6 HC-04 cells. After the antibody incubation at the room temperature for 60 min, cells were washed with 1x DPBS three times. Cells were finally resuspended in 0.3 ml DPBS for applying to BD Accuri™ C6 flow cytometry (BD Biosciences). After FSC-A and SSC-A analysis, cells were gated by the green fluorescence against the original non-transfected HC-04 control.

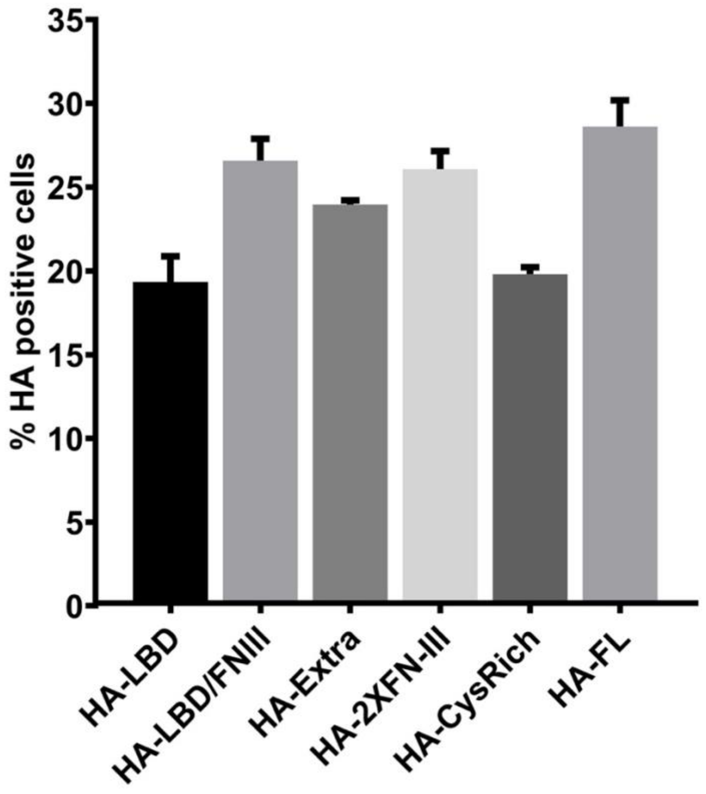

Supplementary figure S2. The size distributions of liver-stage parasites in the EphA2Extra-HC04 clones 4D11, 1C9 and the original HC-04. Each panel represents the liver-stage burden from each of the 8 *P. vivax* isolates (mean ± SD) measured on day 4 post-infection.

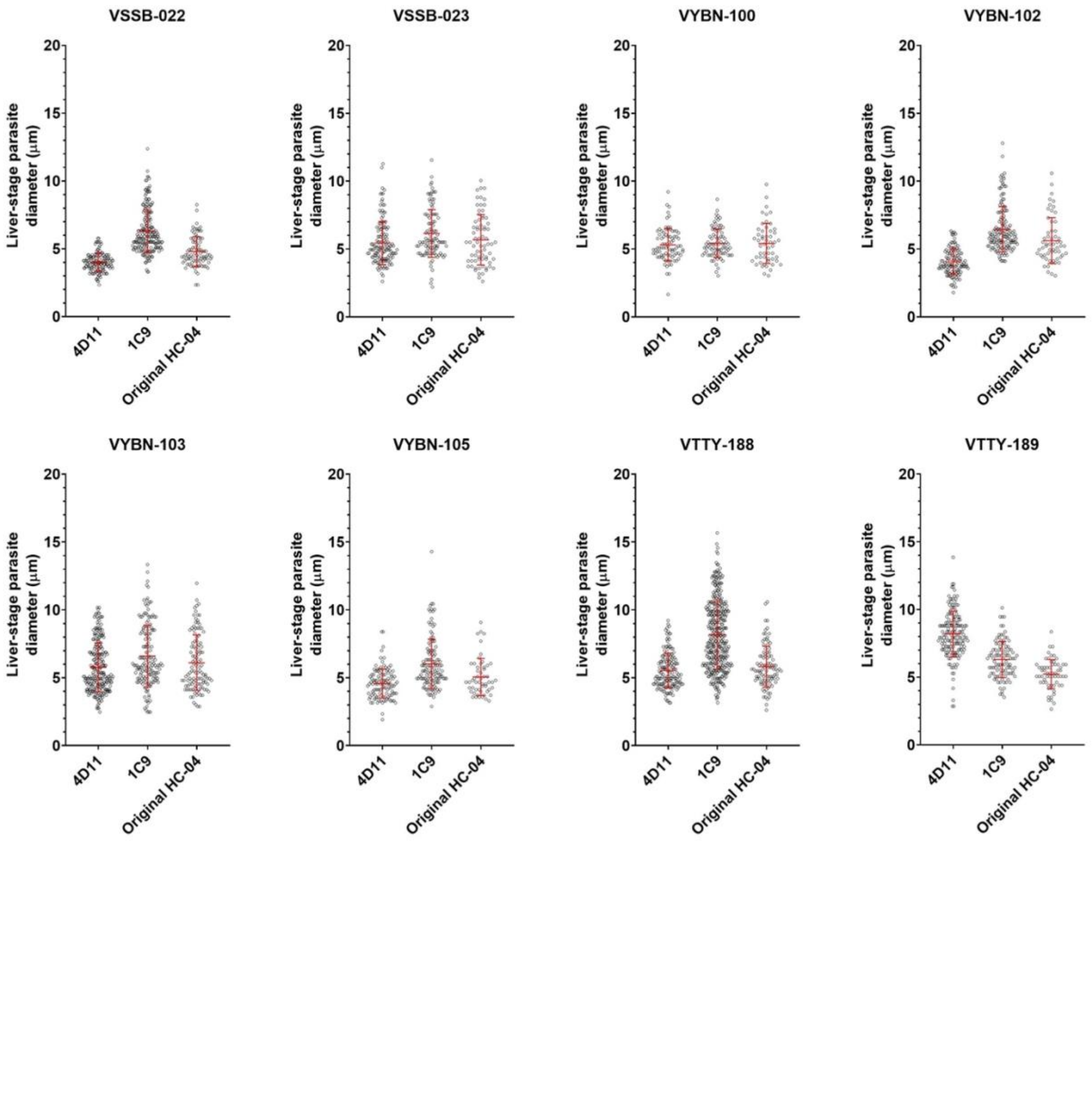

Supplementary figure S3. Representative early *P. vivax* infected EphA2Extra-HC04 (4D11; top, 1C9; bottom) on day 4 post-infection. The liver-stage parasites were labeled with anti-PvUIS4 monoclonal antibody (red). Nuclei were stained with DAPI (blue). The scale bars represent 5 µm.

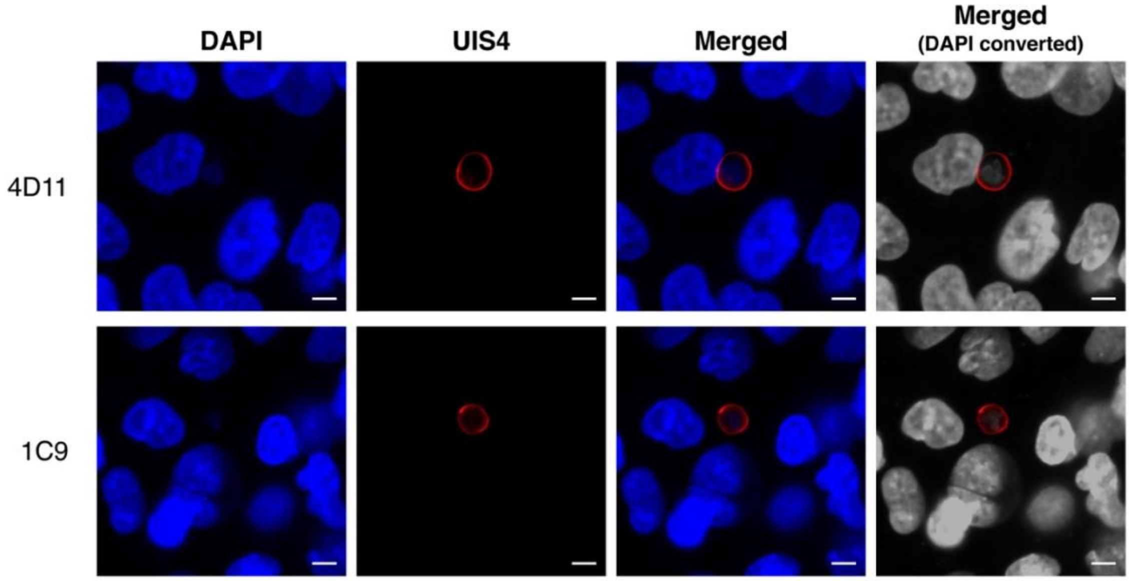

Supplementary figure S4. (A) A full-length western blot of the total cell lysates from HC-04 transfected with different EphA2 constructs. The EphA2 recombinant proteins were detected through the N-terminal HA-tag. The image was separately acquired for each construct. A composite protein marker was applied. (B) A full-length western blot indicated β-actin as the loading control.

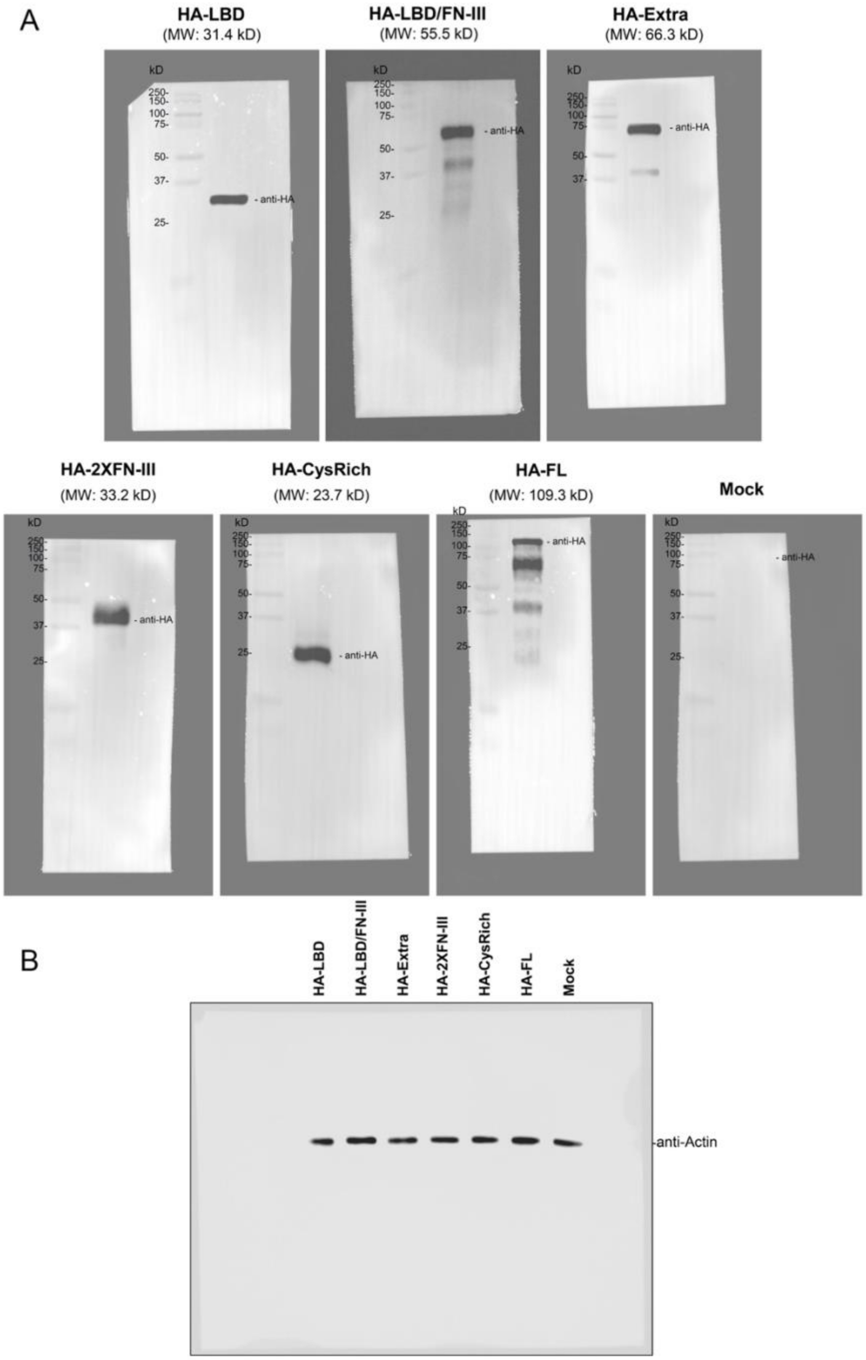

Supplementary figure S5. (A) A full-length of agarose gel electrophoresis indicating the HA-Extra sequence in two clones of the transgenic EphA2Extra-HC04 cell line, resulting in a 2,143-bp amplicon. (B) A full-length western blot of the EphA2Extra-HC04 cell clone lysate with the indicated anti-HA. (C) A full-length western blot of the EphA2Extra-HC04 cell clones with the indicated anti-β-actin was used as the loading control.

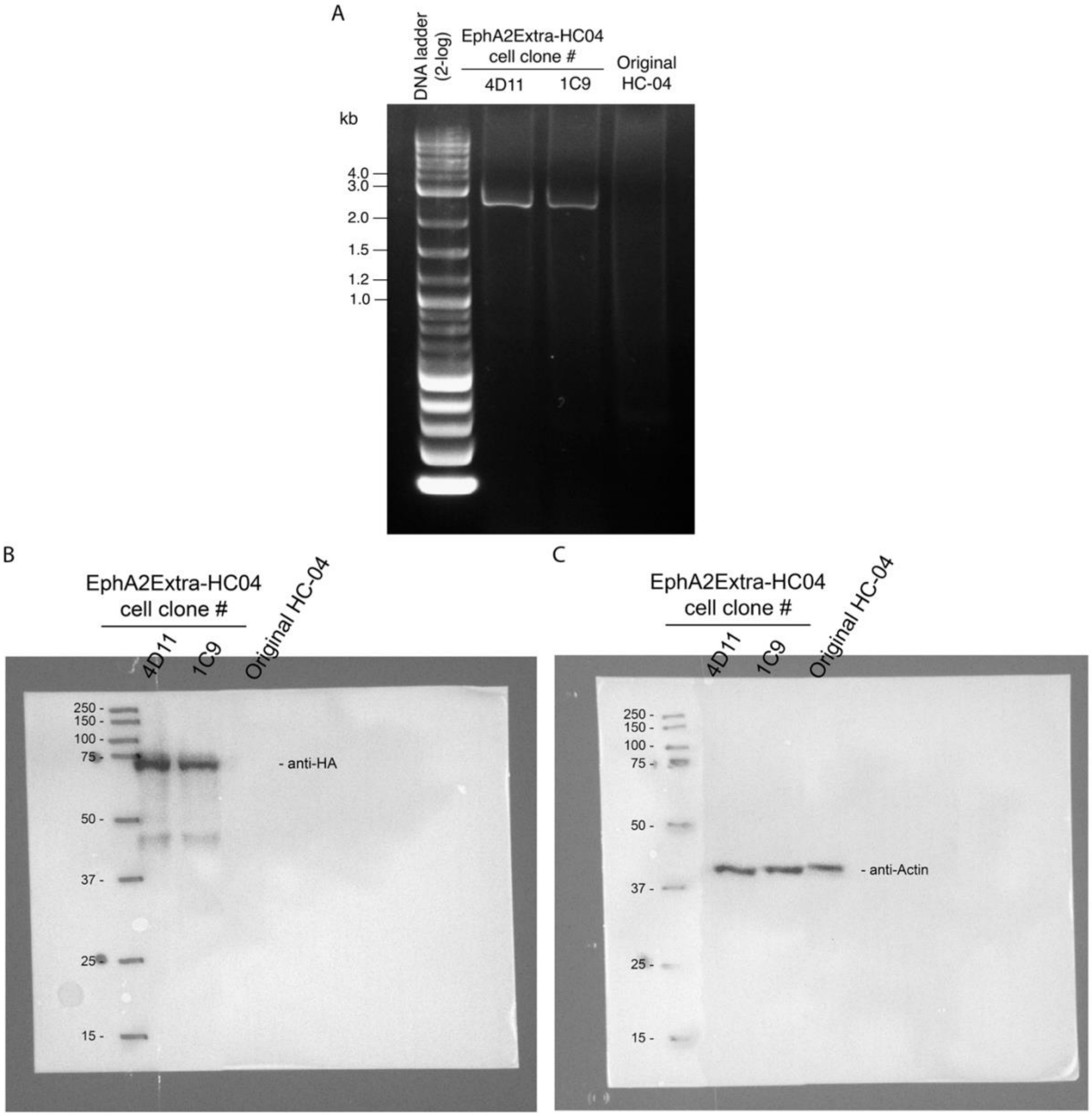

Supplement: Supplementary file 1 — Supplementary Figures. [file 41598_2022_25281_MOESM1_ESM.pdf]
