# Supplementary material for: Overexpression of hepatocyte EphA2 enhances liver-stage infection by Plasmodium vivax
Source: Sci Rep. 2022 Dec 13;12:21542. doi: 10.1038/s41598-022-25281-4 (PMC9746569; doi:10.1038/s41598-022-25281-4)
Supplement: Supplementary file 2 — Supplementary Tables. [file 41598_2022_25281_MOESM2_ESM.pdf]

Overexpression of hepatocyte EphA2 enhances liver-stage infection by *Plasmodium vivax*

Sittinont Chainarin[1,2], Ubonwan Jaihan[1], Parsakorn Tapaopong[1,2], Pinyapat Kongngen[1], Nawapol Kunkeaw[1], Liwang Cui[3], Jetsumon Sattabongkot[1], Wang Nguitragool[1,2*] & Wanlapa Roobsoong[1*]

[1]Mahidol Vivax Research Unit, Faculty of Tropical Medicine, Mahidol University, Bangkok, Thailand, 10400.

[2]Department of Molecular Tropical Medicine and Genetics, Faculty of Tropical Medicine, Mahidol University, Bangkok, Thailand, 10400.

[3]Department of Internal Medicine, Morsani College of Medicine, University of South Florida, Tampa, FL, 33612, USA.

* Corresponding authors: wanlapa.ros@mahidol.edu, wang.ngu@mahidol.edu

Supplementary table

Supplementary Table S1. The mean numbers of liver-stage parasites per well after transient transfection of HC04 with different recombinant EphA2 plasmid constructs.

***P. vivax* isolates**	**Mean number of liver-stage parasites**						
	**HA-LBD**	**HA-LBD/FNlll**	**HA-Extra**	**HA-2XFN-III**	**HA-CysRich**	**HA-FL**	**Mock pDisplay**
VSSB-011	20.0	74.7	97.0	81.3	27.3	40.7	37.0
VTTY-125	23.0	30.5	45.7	22.8	24.8	20.7	18.0
VTTY-134	154.8	172.0	228.7	190.5	165.7	146.8	151.8
VTTY-135	40.7	50.3	65.3	47.3	44.0	43.7	46.3
VTTY-144	13.0	23.3	33.3	28.0	25.7	21.0	18.7
VYBN-040	82.7	111.8	159.0	106.3	80.8	78.8	73.2
VYBN-074	36.7	50.2	64.8	35.3	34.0	25.3	29.0
VYBN-077	18.7	28.0	34.3	26.0	22.8	17.8	17.8
VTTY-169	74.0	74.0	109.0	65.0	53.0	58.0	72.5
VSSB-018	100.0	65.0	78.5	68.0	29.0	45.5	38.5

Supplementary Table S2. The mean numbers of liver-stage parasites per well of EphA2Extra-HC04 clones 4D11 and 1C9.

***P. vivax* isolates**	**Mean number of liver-stage parasites**		
	**4D11**	**1C9**	**Original HC-04**
VSSB-022	159.7	239.7	141.4
VSSB-023	174.0	119.3	109.2
VYBN-100	75.3	79.3	63.2
VYBN-102	151.3	151.3	115.6
VYBN-103	232.3	169.7	148.3
VYBN-105	123.0	134.3	94.2
VTTY-188	178.3	348.7	163.7
VTTY-189	164.0	148.0	137.4

Supplementary Table S3. Primers for amplifying EphA2 fragments. Underlined are BglII and SalI restriction sites.

Constructs	Directions	Oligo-sequences
HA-LBD	Forward	TTATTTAGATCTGAAGTGGTACTGCTGGACTTTGCTG
	Reverse	AATAAAGTCGACCTGCAGCAGCTCGGGGCA
HA-LBD/FNAIII	Forward	TTTATTAGATCTGAAGTGGTACTGCTGGACTTTGC
	Reverse	AATAAAGTCGACGACACTGGCAGTACGGAAGCT
HA-Extra	Forward	TTATTTAGATCTGAAGTGGTACTGCTGGACTTTGCTG
	Reverse	TATTATGTCGACCGGGGACAGCGTCTGGAA
HA-2XFN-III	Forward	AAATAAAGATCTCCCCCCTCCGCCCCACACTA
	Reverse	ATTTATGTCGACCGGGGACAGCGTCTGGAATT
HA-CysRich	Forward	TTTATTAGATCTGGCCTGGCCCACTTCCCTG
	Reverse	ATTTATGTCGACTCGTGTGCAAGGCATCGACG
HA-FL	Forward	TTATTTAGATCTGAAGTGGTACTGCTGGACTTTGCTG
	Reverse	AATAATGTCGACTCACCCCACAGTGTTCACCTGGTCCTT

Supplementary Table S4. Primers for detecting the EphA2Extra-HC04 integrant cells.

Directions	Oligo-sequences
Forward	GGCTTAGCACCTCTCCATCCTCTT
Reverse	ACAGGCACCGATATCCTGGAA
